# Interpersonal early‐life trauma alters amygdala connectivity and sustained attention performance

**DOI:** 10.1002/brb3.684

**Published:** 2017-04-10

**Authors:** Francesca C. Fortenbaugh, Vincent Corbo, Victoria Poole, Regina McGlinchey, William Milberg, David Salat, Joseph DeGutis, Michael Esterman

**Affiliations:** ^1^ Translational Research Center for TBI and Stress Disorders (TRACTS) and Geriatric Research, Education, and Clinical Center (GRECC)VA Boston Healthcare SystemBostonMAUSA; ^2^ Neuroimaging Research for Veterans (NeRVe) CenterVA Boston Healthcare SystemBostonMAUSA; ^3^ Department of PsychiatryHarvard Medical SchoolBostonMAUSA; ^4^ Department of PsychologySchool of Arts and ScienceSouthern New Hampshire UniversityManchesterNHUSA; ^5^ Institute of Aging ResearchHebrew SeniorLifeBostonMAUSA; ^6^ Athinoula A. Martinos Center for Biomedical ImagingCharlestownMAUSA; ^7^ Department of MedicineHarvard Medical SchoolBostonMAUSA; ^8^ Department of PsychiatryBoston University School of MedicineBostonMAUSA

**Keywords:** amygdala, early life trauma, frontoparietal attention network, functional connectivity, middle frontal gyrus, parahippocampal gyrus, sustained attention

## Abstract

**Introduction:**

Interpersonal early life trauma (I‐ELT) is associated with a myriad of functional impairments in adulthood, increased risk of drug addiction, and neuropsychiatric disorders. While deficits in emotional regulation and amygdala functioning are well characterized, deficits in general cognitive functioning have also been documented. However, the neural underpinnings of cognitive dysfunction in adults with a history of I‐ELT and the potential relationship between amygdala‐based functional connectivity and behavioral performance are currently poorly understood. This study examined how I‐ELT affects the cognitive and neural mechanisms supporting sustained attention.

**Methods:**

A total of 66 Veterans (18 with and 48 without a history of I‐ELT) completed a nonemotional sustained attention task during functional MRI.

**Results:**

The individuals with I‐ELT showed significant impairments in sustained attention (i.e., higher error rates, greater response variability). This cohort exhibited increased amygdala functional connectivity with the prefrontal cortex and decreased functional connectivity with the parahippocampal gyrus when compared to those without I‐ELT. These connections were significantly correlated with individual differences in sustained attention performance. Notably, classification analyses revealed that the pattern of amygdala connectivity across the whole brain was able to classify I‐ELT status with 70% accuracy.

**Conclusion:**

These results provide evidence of a lasting negative impact for those with a history of I‐ELT on sustained attention ability. They also highlight a critical role for amygdala functioning in cognitive control and sustained attention for those with a history of I‐ELT, which may underlie the observed attention deficits in clinical assessments and cognitive tests involving both emotional and nonemotional stimuli.

## 1 Introduction

Converging evidence points to a lasting impact of trauma experienced during sensitive periods in childhood (Anda et al., 2006), with developmental trajectories occurring pre/periadolescence irremediably altered by exposure to significant stress (Bergman, Larsen, & Mueller, 1986; Mullen, Martin, Anderson, Romans, & Herbison, 1996; Perry, Pollard, Blakley, Baker, & Vigilante, 1995; Tottenham & Sheridan, 2010). Stressors of an interpersonal nature, like physical/sexual abuse or family violence, occur in 9.5% (Sachs‐Ericsson, Blazer, Plant, & Arnow, 2005) to 32% of the general population (Cougle, Timpano, Sachs‐Ericsson, Keough, & Riccardi, 2010) and are thought to cause enduring emotional or cognitive impairments due in great part to the human agency behind the threat (De Bellis & Zisk, 2014; Perry et al., 1995). It is important to underscore the personal nature of these traumatic events, since they have been documented to lead to more severe symptoms than impersonal traumas like accidents or natural disasters (Perry et al., 1995). Studies of interpersonal early life trauma (I‐ELT) have further revealed increases in reactivity to fear‐conditioning and startle paradigms (Jovanovic et al., 2009, 2011; Ornitz & Pynoos, 1989), decreases in stress regulation (Gunnar, Frenn, Wewerka, & Van Ryzin, 2009), and impaired emotion regulation abilities (McLaughlin, Hatzenbuehler, Mennin, & Nolen‐Hoeksema, 2011). Such behavioral alterations have been associated with increased risk of developing psychopathology in adulthood (Heim & Nemeroff, 2001), especially posttraumatic stress disorder (PTSD) (Lanius, Frewen, Vermetten, & Yehuda, 2010; McKeever & Huff, 2003; Pratchett & Yehuda, 2011).

Stress, which impacts the whole body through the concerted actions of hormones and neurotransmitters (McEwen, 2000), typically begins with and feeds back to the limbic system. This system develops throughout childhood and may be especially affected by exposure to I‐ELT. Animal models provided initial evidence of a lasting impact of ELT on the limbic system (LeDoux, 2000, 2003). Human brain imaging studies subsequently showed that I‐ELT was associated with altered structural integrity in the limbic system (Aas et al., 2012; Andersen et al., 2008; Bremner et al., 1997; Carrion, Weems, & Reiss, 2007; Corbo et al., 2014; Dannlowski et al., 2012; Driessen et al., 2004; Lupien et al., 2011; Tottenham et al., 2010; Veer et al., 2015). Functional imaging studies have further shown that, when processing emotional stimuli (e.g., angry faces), I‐ELT was associated with increased activity in the amygdala (Bremner et al., 2005; Grant, Cannistraci, Hollon, Gore, & Shelton, 2011; Maheu et al., 2010), and with decreased activity of the hippocampus (Bremner et al., 1999; Bremner et al., 2003; Carrion, Haas, Garrett, Song, & Reiss, 2010) and anterior cingulate cortex (Bremner, Vythilingam, Vermetten, Southwick, McGlashan, Nazeer, et al., 2003; Bremner, et al., 2003; Bremner et al., 2004; Mueller et al., 2010). However, these studies have been limited by the consistent use of emotional stimuli, preventing assessments of whether I‐ELT‐related limbic dysfunction potentially impacts more general aspects of cognition.

Importantly, deficits in general cognitive abilities, which are relevant to everyday functioning, have been reported in children (Wilson, Hansen, & Li, 2011) and adults exposed to childhood trauma (Gould et al., 2012). Sustained attention, the ability to maintain focus over a period of seconds to minutes while suppressing distractions, is highly sensitive to stress/arousal (Scholz et al., 2009). Significant evidence points to an impact of I‐ELT on sustained attention ability (De Bellis, Woolley, & Hooper, 2013; DePrince, Weinzierl, & Combs, 2009; Kaplow, Hall, Koenen, Dodge, & Amaya‐Jackson, 2008; Navalta, Polcari, Webster, Boghossian, & Teicher, 2006; Porter, Lawson, & Bigler, 2005; Samuelson, Krueger, Burnett, & Wilson, 2010), yet no study to date has examined how I‐ELT‐related amygdala dysregulation may contribute to impairments in sustained attention or cognitive performance in neutral contexts more broadly. Evidence that amygdala functioning can impact sustained attention in nonemotional contexts can be found in animal studies suggesting that the amygdala serves as a general relevance detector (Gallagher & Holland, 1994; Holland, 2007; Holland & Gallagher, 1999; Holland, Han, & Gallagher, 2000; Sander, Grafman, & Zalla, 2003). Additionally, emerging literature in non‐ELT clinical populations suggests that amygdala functioning may interfere with sustained attention (Fleck et al., 2012).

In both clinical and nonclinical populations, traditional stimulus‐evoked analyses do not typically identify the amygdala as a region involved in sustained attention (Esterman, Noonan, Rosenberg, & DeGutis, 2013; Langner & Eickoff, 2013; Sarter, Givens, & Bruno, 2001; see Fleck et al., 2012 for an exception). However, functional connectivity analyses provide a complementary analytic approach to activation analyses, which focus on brain activation patterns to rare target or error events in sustained attention tasks. Contrasted to activation analyses, functional connectivity takes into account the entire task time series, and are model‐free, capturing cofluctuations in activity not captured by extrinsic events in model‐based activation analyses (Gonzalez‐Castillo et al., 2012). Recent studies using functional connectivity analyses suggest that this approach is sensitive to I‐ELT (Philip et al., 2013; Philip et al., 2014), as well as individual differences in anxiety, intelligence, cognitive ability, and ADHD symptoms (Finn et al., 2015; Fortenbaugh, DeGutis, & Esterman, in press; Kim et al., 2011; Poole et al., 2016; Rosenberg et al., 2016). This approach may thus reveal previously unobserved brain–behavior associations that are relevant to our understanding of cognition and psychopathology.

Based on the promise of this approach, the present study investigated the performance of Veterans with and without exposure to I‐ELT using an innovative sustained attention measure, the gradual onset continuous performance task (gradCPT) (Esterman et al., 2013). We hypothesized that individuals with a history of I‐ELT would show impaired performance on the task. Furthermore, based on findings in the literature indicating that the amygdala may be structurally and functionally altered following a history of I‐ELT, we tested the hypothesis that group‐level differences in amygdala functional connectivity exist across the brain and, furthermore, are related to observed attentional performance differences.

## 2 Methods and Materials

### 2.1 Participants

Sixty‐six participants were recruited from the Translational Research Center for Traumatic Brain Injury and Stress Disorders (TRACTS), a Rehabilitation Research and Development National Center for TBI Research (NCR) at the Veterans Affairs Boston Healthcare System. All participants were deployed Veterans from Operations Enduring Freedom/Operation Iraqi Freedom/Operation New Dawn. The specific characteristics of this cohort have previously been described (Lippa et al., 2015). Exclusion criteria for TRACTS include: (a) history of neurological illness [other than traumatic brain injury (TBI)]; (b) history of seizures; (c) current diagnosis of schizophrenia spectrum or other psychotic disorders (not related to PTSD); (d) current active suicidal and/or homicidal ideation, intent, or plan requiring crisis intervention; or (e) cognitive disorder due to general medical condition other than TBI. Importantly, because our cohort was deployed, exposure to combat and a history of mild traumatic brain injury were not exclusion criteria. The Institutional Review Board of Human Studies Research at the VA Boston Healthcare System approved all research procedures. All participants provided informed consent, and were reimbursed for their time and travel expenses.

History of I‐ELT was assessed using the *Traumatic Life Events Questionnaire* [TLEQ (Kubany et al., 2000)]. Following the criteria used in Corbo et al. (2014), individuals were considered to have I‐ELT (I‐ELT^+^, *N *= 18) if they reported a history of physical abuse, sexual abuse, or family violence before 18 years of age, in addition to a fearful (A2) response (APA, 2013). Control participants (I‐ELT^−^, *N *= 48) were individuals without this history. Although some control participants (*N *= 26) did report exposure to a traumatic event of a noninterpersonal nature before 18 years old (e.g., earthquake or motor vehicle accident), they were included in the control group if they did not report significant distress (no A2 reaction or PTSD diagnosis). To ensure that group differences were specifically due to the interpersonal quality of the traumatic event, we further examined I‐ELT^+^ versus the I‐ELT^−^ who had been exposed to a noninterpersonal trauma before 18 years of age.

### 2.2 Clinical assessment

As part of the standard TRACTS protocol, all participants completed clinical interviews regarding their deployment experiences, as well as pre‐ and postdeployment traumatic experiences. In order to establish consensus on the diagnoses, three doctoral‐level psychologists reviewed all clinical interviews. During this assessment period, PTSD diagnosis and severity was evaluated using the *Clinician‐Administered PTSD Scale* (CAPS; Blake et al., 1995). Severity of PTSD in the past month was computed by summing the frequency and intensity of symptoms in the three main clusters (Flashbacks and Intrusive Memories; Avoidance and Emotional Numbing; and Hyperarousal). Predeployment assessment of PTSD on the CAPS was used to help confirm assessment of I‐ELT. History of TBI was assessed using the *Boston Assessment of TBI‐Lifetime* (BAT‐L; Fortier et al., 2013). The BAT‐L covers events pre‐, during, and post‐military deployment, allowing for computation of the total number of mild TBIs across the lifetime. Because of the impact of physical trauma to the brain, we used the BAT‐L to exclude participants reporting a history of moderate/severe TBIs, while recording the number of mild TBIs experienced as a statistical control. Combat exposure, a proxy for severity of military trauma, was assessed using the combat scale of the *Deployment Risk and Resilience Inventory* (DRRI; Vogt, Proctor, King, King, & Vasterling, 2008). Severity of symptoms of depression was documented using the depression scale of the *Depression, Anxiety and Stress Scale* (DASS‐d; Koenig, Jacob, & Haber, 2009). DASS results for one I‐ELT^−^ participant were not available. This participant was excluded from ANCOVA analyses using clinical measures as covariates. All TRACTS participants completed the MRI session at the end of the testing day.

### 2.3 Behavioral paradigm and stimuli

**Figure 1:**
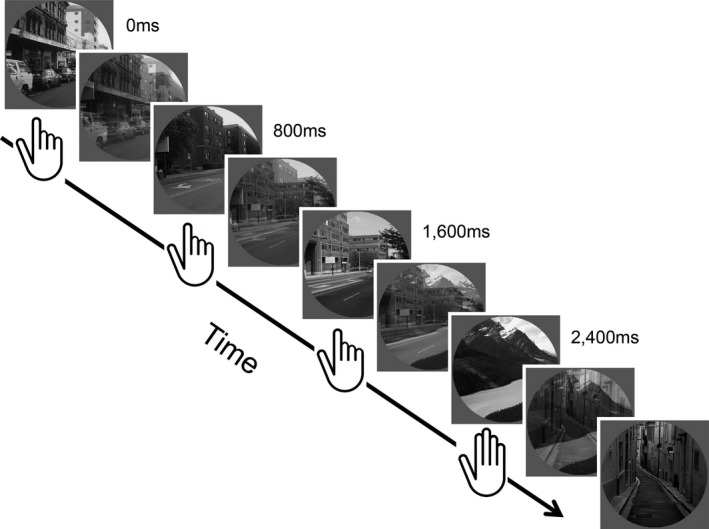
Behavioral paradigm. Illustration of the trial sequence in the gradual onset continuous performance task (gradCPT). This figure shows images linearly transitioning from one to the next over each 800 ms interval. Images were shown one after the next for the entire 8‐minute run. Participants were asked to press a button every time they detected a new city image and withhold pressing whenever they detected a rare mountain image (10% trials). The mixed images show the adjacent images at 50% coherence levels

The gradCPT is a go/no‐go continuous performance task that uses a not‐X design similar to other continuous performance tasks used in neuropsychological assessments such as the Connors CPT task (Conners, 1994) and the Sustained Attention to Response Task (SART; Robertson, Manly, Andrade, Baddeley, & Yiend, 1997). Participants were instructed to press a button for each city scene they detected (frequent nontarget), and withhold response to each detected mountain scene (infrequent target). The paradigm contains 20 round, gray‐scale photographs of 10 mountain scenes and 10 city scenes that gradually transitioned from one to the next over 800 ms using a linear pixel‐by‐pixel interpolation (see Figure 1). These scenes are randomly presented with 10% mountain and 90% city images, without allowing identical scenes to repeat on consecutive trials. Response accuracy was emphasized without reference to speed. However, given that the next stimulus would replace the current stimulus in 800 ms, a response deadline was implicit in the task. Images were viewed through a mirror in the scanner bore projected via a back projection screen.

Prior to scanning, participants were familiarized with each of the 20 scene images and were given a 1–2 min practice of the task. Within the scanner, participants performed a single 8‐min run (600 trials), which was the final functional task in the more extensive TRACTS protocol.

Following the standard method used in previous studies of the gradCPT (Esterman, Reagan, Liu, Turner, & DeGutis, 2014; Esterman et al., 2013; Fortenbaugh et al., 2015), all responses were logged during the course of the experiment. Following completion, button presses were assigned to individual trials using an iterative algorithm. Reaction times were calculated relative to the beginning of each image transition, such that reaction times (RT) of 800 ms indicate a button press at the moment image N was 100% coherent and not mixed with other images. A shorter RT indicates that the current scene was still in the process of transitioning from the previous, and a longer RT indicates that the current scene was in the process of transitioning to the subsequent scene. So, for example, an RT of 720 ms would be at the moment of 90% image N and 10% image N−1, and so forth.

Once the response algorithm was run, mean reaction times (RT) and reaction time variability scores were calculated from the majority of trials in which participants responded correctly to city scenes (correct commission trials). Reaction time variability was calculated using the coefficient of variation (CV) or the standard deviation of the RT divided by the mean. Two types of errors were considered: commission errors (CE), where participants press to target mountain trials, and omission errors (OE), where participants failed to press to nontarget city trials. Using standard signal detection analyses, these errors were used to calculate both d’, a measure of discrimination ability, and criterion, a response bias measure that reflects the bias of a participant to press to a trial image in the case of uncertainty. To calculate these measures, hit rates were defined as 1 ‐ CE rate, or the percentage of targets for which participants correctly withheld a response. False alarm rates were defined by the OE rate or the percentage of nontargets for which participants incorrectly withheld responses. All participants had hit rates below 100% and false alarm rates greater than 0%. Recent studies have shown that d’ and CV scores reflect a participant's sustained attention ability on the gradCPT, whereas criterion and mean RT best reflect the strategy used (Fortenbaugh et al., 2015). All participants met the performance criteria for inclusion used in previous studies of this task of no periods of 30 s or greater without a response (Fortenbaugh et al., 2015).

### 2.4 MRI acquisition, processing and analysis

Scanning was performed at the Neuroimaging Research for Veterans (NeRVe) center of the VA Boston Healthcare hospital on a 3T Siemens MAGNETOM Trio system. The functional scan was collected using a 32‐channel head coil while two anatomical magnetization‐prepared rapid gradient‐echo (MP‐RAGE) structural scans were obtained with a 12‐channel coil. Functional scans were acquired using one whole‐brain echo‐planar T2*‐weighted sequence with the following parameters: repetition time (TR) = 2000 ms, echo time (TE)  = 30 ms, flip angle = 90º, 248 volumes, acquisition matrix = 64 × 64, in‐plane resolution 3.0 × 3.0 mm^2^, slice thickness 3.75 mm. For structural brain images, two MP‐RAGE T‐1 structural scans were acquired (TR = 2530 ms, TE = 3.32 ms, flip angle = 7º, 256 × 256 × 176, voxel‐size = 1 mm^3^) and averaged to increase signal‐to‐noise ratio. Structural scans were inspected immediately after acquisition on the operator console by the biomedical technician in radiology and a member of the research study staff for motion artifacts and repeated if necessary. All structural images were then processed using standard FreeSurfer and Analysis of Functional NeuroImages (AFNI; RRID:SCR_005927) pipelines (Cox, 1996; Fischl, Sereno, & Dale, 1999; Fischl et al., 2004).

### 2.5 fMRI preprocessing and general linear model

Functional MRI data was processed using AFNI (AFNI; Cox, 1996); and custom routines written in Matlab (Mathworks Inc., Natick, MA, USA, RRID:SCR_001622). Preprocessing steps included slice‐time correction, motion correction using a 6‐parameter, rigid body, least‐squares alignment procedure, spatial smoothing to an 8‐mm FWHM Gaussian kernel, automated coregistration and normalization of anatomical and functional volumes to Talairach space, and scaling of functional dataset values to percent signal change. Data from individual participants were submitted to a first‐level general linear model (GLM) analysis in order to regress out variance in the time series associated with the six motion parameters, and three nuisance regressors. These included cerebral spinal fluid (CSF), white matter, and the global signal time series. Functional connectivity and decoding analyses were completed on the residual time series from this first‐level GLM.

### 2.6 ROI selection

From the Talaraiched structural images, the TT_N27 atlas in AFNI, based on the Eickhoff–Zilles cytoarchitectonic probabilistic atlas developed in the SPM Anatomy Toolbox (Eickhoff et al., 2005), was used to anatomically identify our a priori primary regions of interest (ROI): the left and right amygdala for each participant. To assess group‐level differences in functional connectivity, a bilateral amygdala ROI was used. In the following prediction analyses, the left and right amygdala were treated as separate features. This structural atlas was also used to divide the brain (cortical, subcortical, and cerebellum) into 116 anatomically defined ROIs in the subsequent classification analysis.

### 2.7 Statistical analyses

#### 2.7.1 Between‐group analyses

Given the unequal sample sizes, between‐group behavioral and amygdala connectivity comparisons were conducted using nonparametric Mann–Whitney U*‐*tests.

#### 2.7.2 Functional connectivity analyses

For whole‐brain voxel‐level functional connectivity analyses, the average time series extracted from the bilateral amygdala ROI was correlated with the time series of all other voxels. The resulting Pearson r‐values were converted to Fisher z‐scores to use in the between‐group analyses. All resulting statistical maps were corrected for multiple comparisons using a new and appreciably more conservative voxel‐cluster Monte Carlo‐type α simulation than the previous standard Gaussian model in AFNI. First, the AFNI 3dFWHMx function was run using the spatial autocorrelation function (ACF) option to estimate the smoothness of the data using a mixed Gaussian plus mono‐exponential model to generate random noise fields. The estimated parameters were then used with the 3dClustSim function, again using the ACF option, to estimate the minimum cluster sizes needed. Based on results from this analyses, between‐group connectivity comparisons cluster‐corrected thresholds for *p *<* *.05 were given by nominal *p *=* *.01 and cluster size ≥ 77.

#### 2.7.3 Decoding behavior

To determine if amygdala connectivity patterns predicted behavioral performance, multiple linear regression analyses were completed using a leave‐one‐subject‐out (LOSO) approach (Esterman, Tamber‐Rosenau, Chiu, & Yantis, 2010), which maintains independence between the training and testing datasets. Here, separate group‐level functional connectivity difference analyses using the left and right amygdala ROIs as seed regions were rerun 66 times, each time leaving one participant out. The two significant clusters (as found in the initial between‐group analysis) with the bilateral amygdala ROI were redefined for each analysis setting the nominal *p *=* *.01, meaning that the exact number of voxels in each cluster varied across the 66 permutations (range = 26–226 voxels). This resulted in a total of four features that were used in the linear regression analysis (i.e., the left and right amygdala connections to the two clusters were calculated separately). Here, the average functional connectivity z‐scores for each of the 66 participants were extracted from each of the four clusters. The average z‐scores for the 65 participants in the training set were then used as independent variables in a multiple linear regression to predict either their d’ or CV scores (i.e., the two behavioral measures primarily related to sustained attention ability). The coefficients of these two models were then applied to the functional connectivity z‐scores of the one participant in the test dataset to obtain a predicted performance value (d’ or CV). Once this procedure had been completed 66 times with each participant left out exactly once, the predicted performance scores were correlated with the observed performance scores (e.g., predicted d’ vs. observed d’) to test the performance of the model using Pearson‐r correlation analysis.

#### 2.7.4 Decoding I‐ELT status

The final analysis used a whole‐brain ROI‐level classification analysis to determine if I‐ELT status could be predicted based on amygdala connectivity patterns. Classification was completed using discriminant analysis in Matlab with functions from the Statistics and Machine Learning toolbox and custom written functions. A naïve Bayes classifier was used, estimating the diagonal covariance matrix with multivariate normal densities. This algorithm assumes features are conditionally independent given the class. While this assumption is most often not met, this type of classifier has been found to be effective at classification in a variety of settings, including probabilistic diagnosis (Hilden, 1984) and when using small sample sizes (Hand & Yu, 2001).

For this ROI‐level analysis, the TT_N27 atlas was used to divide the brain into 116 ROIs. The average time‐series for each ROI was correlated with the left and right amygdala time series and the resulting correlation values were Fisher z‐scored. This resulted in 232 potential features for the LOSO classification analysis. Using the LOSO approach, each participant was left out of the training dataset once. In each LOSO iteration, a model was built from N features across the 65 participants in the training dataset, were N was varied from 1 to 66 possible features. The maximum feature set of 66 was chosen to not exceed the number of instances (subjects). While in traditional modeling analyses, increasing the number of independent variables inevitably lead to increases in the amount of variance explained and model over‐fitting, the same is not true when the LOSO approach is utilized. Indeed, increasing the number of features here leads to modeling of noise in the training set which can in turn decrease prediction ability on the test dataset (Hawkins, 2004). Feature selection was done by finding the N features with the greatest between‐group separation (I‐ELT^+^ vs. I‐ELT^−^) based only on the 65 participants used in the training dataset, thus maintaining independence between the training and testing datasets. Thus, for a given feature set size, the features included in the model could vary across participants. For each feature set, the z‐scores from the 65 participants in the training set were used to build a classification model. This model was then applied to the z‐scores from the participant left out (test dataset) from the same feature set to predict their I‐ELT status (0 or 1). For every participant, accuracy of classification was assessed for (1) overall accuracy, (2) sensitivity (proportion of I‐ELT^+^ participant correctly classified), and (3) specificity (proportion of I‐ELT^−^ participants correctly classified).

## 3 Results

### 3.1 Behavioral differences

**Figure 2:**
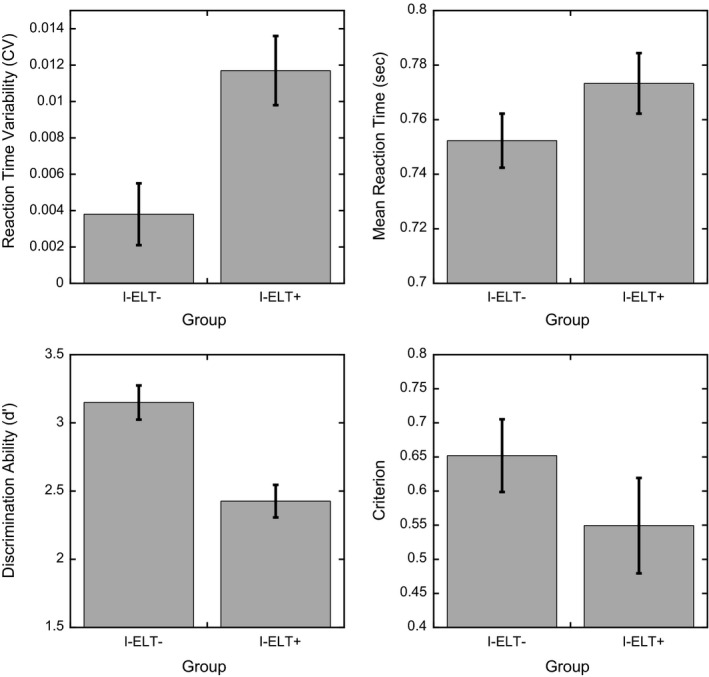
Behavioral performance as a function of I‐ELT group for the four primary measures of interest related to ability (discrimination ability and reaction time variability; left panels) and strategy (criterion and mean reaction time; right panels). Reaction time variability is defined by the coefficient of variation (CV). Error bars show ±1 S.E.M.

As seen in Table 1, the groups were matched in terms of age, gender, estimated IQ, PTSD diagnosis and severity, combat exposure, and depression. Analyses of group‐level behavioral differences are summarized in Table 2 and Figure 2. Collectively, I‐ELT^+^ participants showed deficits in sustained attention ability (i.e., higher error rates and lower d’ scores) compared to the I‐ELT^−^ control group. These findings remained significant after simultaneously controlling for current PTSD and depression symptom severity, number of lifetime mild TBIs, and age using an ANCOVA with the four additional independent variables included as continuous covariates (I‐ELT factor: *F*_1,59_ = 9.427, *p *=* *.003). None of the covariates were found to be significant in this model (*p *>* *.22 for all three; see Table 3). We found significantly more omission errors in the I‐ELT^+^ group and a trend toward more commission errors. From the distribution of omission errors, we found no difference in the overall omission durations, defined by the number of consecutive nontarget/city trials for which participants failed to respond, but observed a significant increase in the number of discrete omission error periods (Table 2). Thus, the increase in omission error rate was not due to extended periods of nonresponsiveness in the I‐ELT^+^ group but rather increased rates of intermittent failures to maintain task performance. Furthermore, the reaction times to nontarget/city images were significantly more variable across the whole task in the I‐ELT^+^ group, reflected in the CV measure. In contrast, no group level differences were found for either criterion or the mean reaction time.

**Table 1:** Participant Demographics. This table shows the means and ±1 standard deviation for each group, along with the corresponding between‐group statistical comparison

	I‐ELT^−^ (*N *= 48)	I‐ELT^+^ (*N *= 18)	Statistic (*df* = 64)
Age (years)	31.56 ± 7.88	34.22 ± 7.98	*t *=* *1.22, *p *=* *.23
Sex (M:F)	45:3	17:1	χ^2^ = 0.01, *p *=* *.92
IQ	104.56 ± 10.94	100.29 ± 14.56	*t *=* *1.26, *p *=* *.21
PTSD Dx (Y:N)	24:24	11:7	χ^2^ = 0.65, *p *=* *.42
CAPS current	40.15 ± 24.53	50.61 ± 23.57	*t *=* *1.56, *p *=* *.12
Depression	6.35 ± 7.90	9.53 ± 7.73	*t *=* *1.43, *p *=* *.16
Combat exposure (months)	16.38 ± 11.93	15.56 ± 10.32	*t *=* *0.26, *p *=* *.80
Number of lifetime mTBI	1.31 ± 1.36	1.17 ± 1.38	*t *=* *0.39, *p *=* *.70

I‐ELT, interpersonal early life trauma; mTBI, mild TBI.

**Table 2:** Behavioral performance results. This table shows the means and ±1 standard error of the mean for each group and the between group statistical results from Mann–Whitney U tests with the z‐statistic shown. Coefficient of variation is the normalized reaction time variability measure (see Methods)

	I‐ELT^−^ (*N *= 48)	I‐ELT^+^ (*N *= 18)	Statistic
Discrimination ability (d’)	3.150 ± 0.125	2.426 ± 0.195	*Z*(64) = −2.980, *p *=* *.003
Criterion	0.652 ± 0.053	0.549 ± 0.114	*Z*(64) = −0.691, *p* = .490
Reaction time (sec)	0.752 ± 0.010	0.773 ± 0.018	*Z*(64) = −0.648, *p* = .517
Coefficient of variation	0.181 ± 0.006	0.215 ± 0.012	*Z*(64) = −2.433, *p* = .015
Commission error rate	0.203 ± 0.019	0.293 ± 0.045	*Z*(64) = −1.490, *p* = .136
Omission error rate (OE)	0.034 ± 0.010	0.068 ± 0.019	*Z*(64) = −2.722, *p* = .006
Number OE lapses	14.354 ± 3.626	27.389 ± 8.205	*Z*(64) = −2.618, *p* = .009
Average duration OE lapse (trials)	1.043 ± 0.066	1.360 ± 0.234	*Z*(64) = −1.641, *p* = .101

I‐ELT, interpersonal early life trauma.

**Table 3:** Behavioral performance results. Statistical results from ANCOVAs on the four primary behavioral performance factors. The ANCOVAs include participant age in years, current PTSD severity score (CAPS), current depression severity (DASS_d), and the number of lifetime mild TBIs (mTBI) as continuous covariates

	D′	Criterion	RT	CV
I‐ELT	*F*_1,59_ = 9.427, *p* = .003	*F*_1,59_ = 0.763, *p* = .386	*F*_1,59_ = 0.402, *p* = .529	*F*_1,59_ = 8.527, *p* = .005
Age	*F*_1,59_ = 0.059, *p* = .809	*F*_1,59_ = 0.449, *p* = .506	*F*_1,59_ = 3.138, *p* = .082	*F*_1,59_ = 0.072, *p* = .789
CAPS	*F*_1,59_ = 0.556, *p* = .459	*F*_1,59_ < 0.001, *p* = .991	*F*_1,59_ = 1.039, *p* = .312	*F*_1,59_ = 0.295, *p* = .589
DASS_d	*F*_1,59_ = 0.177, *p* = .676	*F*_1,59_ = 0.227, *p* = .601	*F*_1,59_ = 0.023, *p* = .879	*F*_1,59_ = 0.036, *p* = .849
mTBI	*F*_1,59_ = 1.523, *p* = .222	*F*_1,59_ = 0.974, *p* = .386	*F*_1,59_ = 0.755, *p* = .388	*F*_1,59_ = 0.743, *p* = .392

CV, coefficient of variation; RT, reaction times; I‐ELT, interpersonal early life trauma.

As noted in the Participants section, the I‐ELT^−^ group included individuals who had been exposed to a noninterpersonal trauma before 18 years of age (*N *= 26/48). To examine if the behavioral differences we observed across the I‐ELT^+^ and I‐ELT^−^ group are specific to trauma of an interpersonal nature, we compared the performance of the 26 I‐ELT^−^ participants with a history of noninterpersonal trauma before 18 years of age to the I‐ELT^+^ group and the remaining 22 I‐ELT^−^ participants with no history of trauma before 18 years of age. For all behavioral performance measures, the same pattern of differences was observed for the I‐ELT^+^ group compared to the I‐ELT^−^ with or without a history of noninterpersonal trauma before age 18 years. In contrast, no difference was found for any behavioral measure when comparing the I‐ELT^−^ participants with versus those without a history of noninterpersonal trauma before 18 years of age. Collectively, these results demonstrate that deficits in sustained attention ability in this sample are specific to early life traumas of an interpersonal nature. Thus, for all of the following imaging analyses, no differentiation was made of participants in the I‐ELT^−^ group who had, or had not, been exposed to a noninterpersonal trauma before 18 years of age.

### 3.2 Functional connectivity of the amygdala

**Figure 3:**
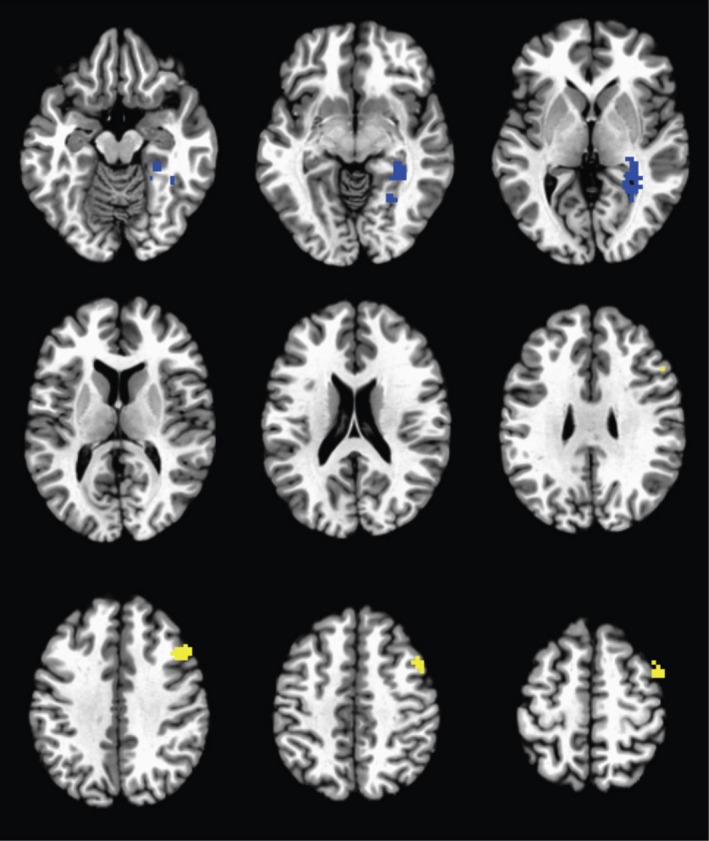
Group‐level differences in functional connectivity with the bilateral amygdala seed. Images are cluster‐corrected and threshold to *p* < .05. The right parahippocampal gyrus demonstrated decreased functional connectivity (blue) and the right middle frontal gyrus demonstrated increased functional connectivity (yellow) in the I‐ELT ^+^ group relative to the I‐ELT ^−^ group

Group‐level differences in bilateral amygdala connectivity were found in two clusters (Figure 3). A decrease in functional connectivity was found between the amygdala and the right parahippocampal gyrus for the I‐ELT^+^ group relative to the I‐ELT^−^ group. In contrast, an increase in functional connectivity was observed between the amygdala and the right middle frontal gyrus in the I‐ELT^+^ group. We note that the same two clusters survived when a more conservative threshold was used, with cluster‐corrected thresholds for *p *<* *.05 given by nominal *p *=* *.005; cluster size ≥ 48. At an even more conservative threshold using a nominal *p *=* *.001 (cluster size > 17), only the right middle frontal gyrus cluster survived thresholding. However, as seen below, the additional analyses completed next further demonstrate that information distinguishing the I‐ELT^+^ and I‐ELT^−^ groups is seen in the right parahippocampal‐amygdala connectivity patterns.

To test whether these effects are driven by I‐ELT or rather common I‐ELT comorbidities, a whole‐brain ANCOVA was run comparing the bilateral amygdala connectivity patterns across the I‐ELT^+^ and I‐ELT^−^ groups with age, current PTSD severity, current depression severity, and number of lifetime mild TBIs as continuous covariates. Results of this analysis showed the same two clusters and differences in connectivity patterns across the I‐ELT groups after controlling for all covariates, indicating that participant age and the additional clinical factors cannot account for the amygdala connectivity differences.

### 3.3 Functional connectivity patterns predict behavioral performance

**Figure 4:**
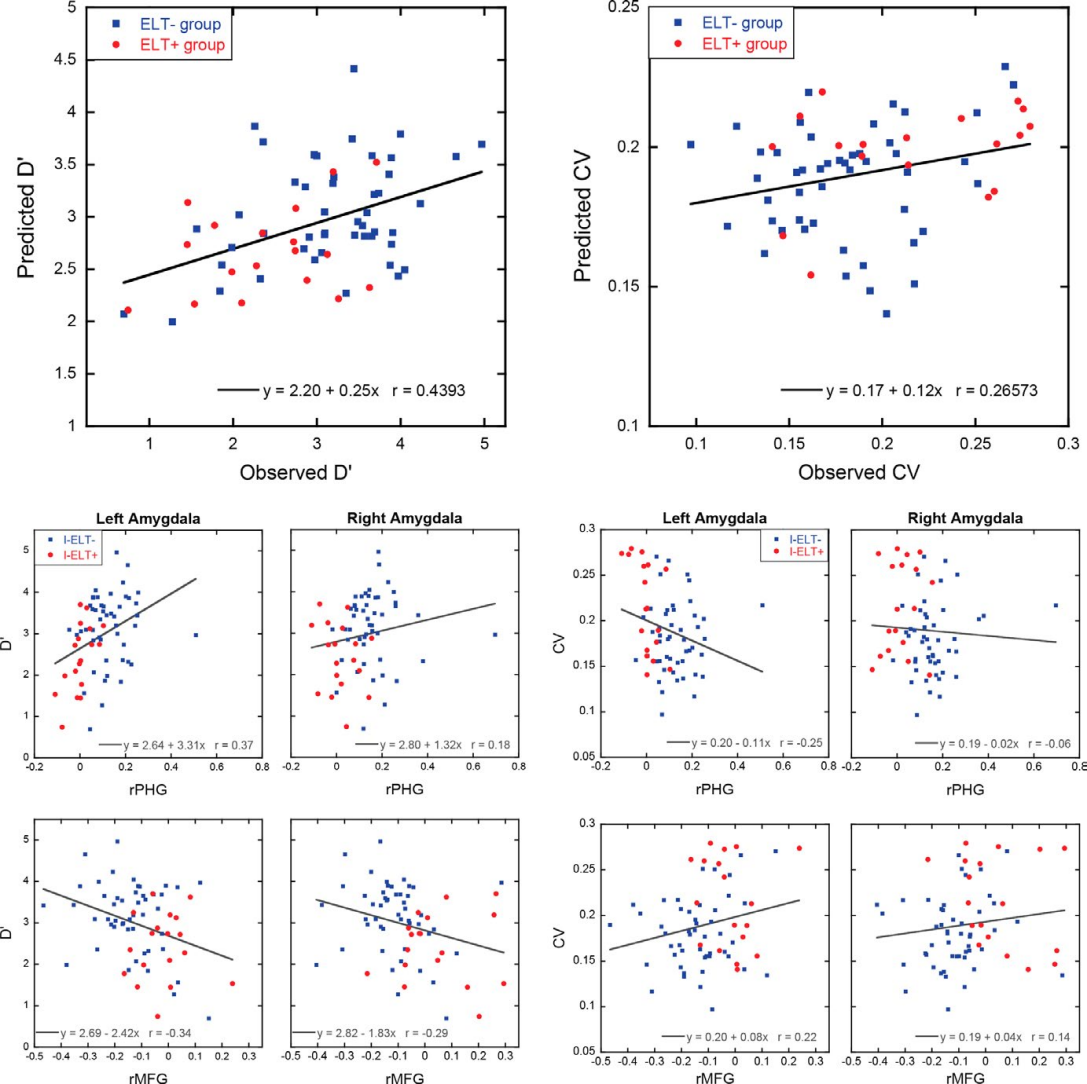
Scatterplots in the two top panels showing the relationship between the observed and predicted discrimination ability (*d’*; left panel) and the observed and predicted reaction time variability (CV; right panel) for each participant using LOSO multiple linear regression approach with the four amygdala connectivity clusters as predictors. While the models were built without regard to I‐ELT status, for illustrative purposes I‐ELT ^+^ participants are shown as red circles while I‐ELT ^−^ participants are shown as blue squares. The bottom panels show first‐level correlations for each of the four amygdala connections used in the multiple linear regression analysis with *d’* (left) and CV (right). The regression lines in these bottom panels are for illustrative purposes only, and were calculated using the entire dataset without cross‐validation

Leave‐one‐subject‐out (LOSO) multiple linear regression analyses were completed to test if the functional connectivity differences predict behavioral performance. Figure 4 (top panels) shows the correlation between the observed and predicted d’ from the LOSO multiple linear regression model. A significant positive correlation was observed [*r *=* *.4393, *p *=* *.0002], indicating that patterns of functional connectivity between the left and right amygdala to the right parahippocampal gyrus and the right middle frontal gyrus predicted individual differences in sustained attention ability. Note that these ROIs were defined based on group‐level differences in history of I‐ELT, and thus were agnostic to individual performance measures (d’). We used the same approach to also predict reaction time variability (CV) scores (see Figure 4). Although weaker, a significant positive correlation was found between the observed and predicted reaction time variability scores [*r *=* *.266, *p *=* *.031]. In order to illustrate the four connections used in the cross‐validated multiple regression procedure, we also plot in the bottom half of Figure 4 the first‐order correlations of each connection with the two behavioral measures of interest. Without using the LOSO cross‐validation procedure, across the full sample for d’ the left amygdala connection with the right parahippocampal gyrus and right middle frontal gyrus show the strongest relationship (*r *=* *.37, *p *=* *.002 and *r *=* *−.34, *p *=* *.005, respectively). For the right amygdala connections, only the connection with the right middle frontal gyrus showed a significant relationship with d’ (*r *=* *−.29, *p *=* *.017). In contrast, of all four amygdala connections only the left amygdala to right parahippocampal gyrus showed a significant relationship with CV (*r *=* *−.25, *p *=* *.04; all others *p *≥* *.08). Of note, all of these correlations comparing amygdala connections to behavioral performance independently show the relationship across the entire dataset without using cross‐validation. These first‐order correlations demonstrate that when predicting behavioral performance, prediction ability is improved by combining information across all four features.

To test whether including current PTSD or depression severity, or the age of participants, as additional independent variables improved prediction ability, the same LOSO multiple linear regression approach was utilized with seven independent variables (the four connectivity scores, CAPS current scores, DASS depression severity score, and the age of participants in years). Results of this analysis showed no improvement in prediction ability regardless of whether one, two, or all three additional variables were included as predictors in the models (d’: 0.391 < *r *<* *.430; CV: 0.184 < *r *<* *.249).

### 3.4 Amygdala functional connectivity predictions of I‐ELT status

**Figure 5:**
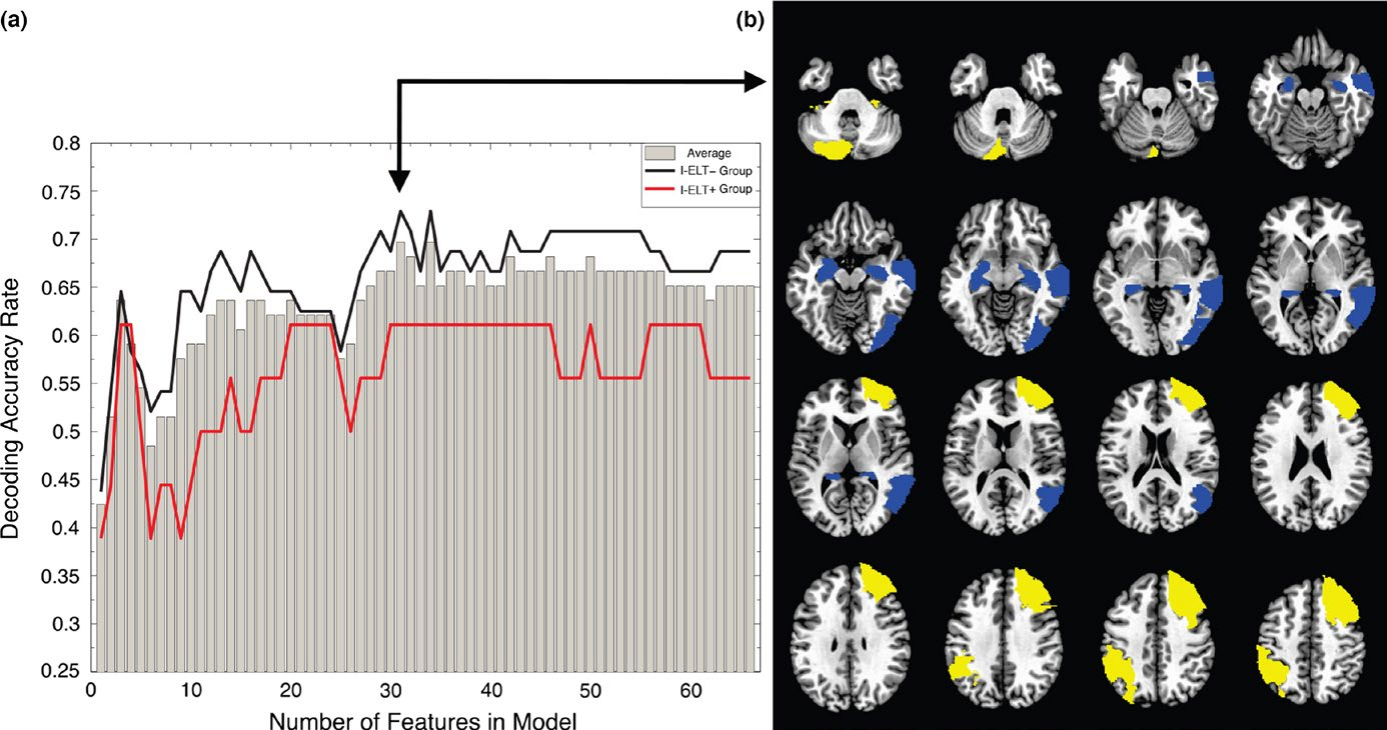
Classification of I‐ELT status based on amygdala connectivity patterns. (a) Decoding accuracy as a function of the number of features (i.e. ROIs) in classification model, illustrating the saturation in decoding accuracy beyond 31 features. The gray bars show the accuracy rate across all participants. The red line shows the accuracy rate for the I‐ELT ^+^ group while the black line shows the accuracy rate for the I‐ELT ^−^ group. (b) Overlay masks showing the ROIs that were chosen in 66/66 of the 31‐feature models across all participants (16 connections from 12 unique ROIs). Blue regions show ROIs with decreased amygdala connectivity in the I‐ELT ^+^ group relative to the I‐ELT ^−^ group, while yellow regions show ROIs with increased amygdala connectivity in the I‐ELT ^+^ group relative to the I‐ELT ^−^ group

A whole‐brain ROI‐level classification analysis was completed to determine whether amygdala functional connectivity with the anatomically parcellated whole brain (see ROI Selection) is sensitive to differences between participant groups and if these differences can be used to predict I‐ELT status. This provides another way to assess the importance of amygdala‐parahippocampal and amygdala‐prefrontal connectivity to I‐ELT status, identify other amygdala connections important to I‐ELT status, and determine how specific/sensitive task‐related amygdala connectivity is in predicting I‐ELT status. Figure 5 shows the decoding accuracy as a function of the number of features used in the model. The best model used 31 features with an overall accuracy rate of 70% and prediction accuracy saturating for larger feature sets with no increase in the average prediction accuracy beyond this point, most likely due to increased modeling of noise in the training data set with greater feature numbers. The 31 features model has a specificity of 73%, correctly classifying 35/48 I‐ELT^−^ participants. The sensitivity of the model is 62%, correctly classifying 11/18 I‐ELT^+^ participants. The diagnostic odds ratio of the model is 3.55.

To determine if the overall accuracy of the 31‐feature model was significantly above chance, Monte Carlo permutation testing was completed. Here, the null‐model was that the pattern of z‐scores is not related to I‐ELT status. For this, the 31‐feature model procedure was repeated 1000 times. Prior to each iteration, the I‐ELT status labels (0's or 1's) were randomly shuffled. The entire 66 LOSO fold was then repeated for each shuffled label set. After each participant's status was classified, the average accuracy of the model across participants was calculated. After 1000 iterations, the distribution of accuracy scores that would happen by chance was calculated. The significance of the 31‐feature model, the p‐value, was then calculated as the proportion of the shuffled models that had an overall accuracy rate equal to or greater than the observed accuracy rate. Results of this showed only 28/1000 random models that met this criterion (*p *=* *.028).

For any individual participant left out, the features used in the model could vary. Figure 5 shows the 12 ROIs selected as features in all 66 participant models with the color indicating whether the ROIs showed increased (yellow) or decreased (blue) connectivity with the amygdala seed ROIs in the I‐ELT^+^ relative to the I‐ELT^−^ group. These 12 ROIs contributed 16 features to the model as follows: two ROIs connected to the left amygdala only, six ROIs were connected to the right amygdala only, and the remaining four ROIs contributed eight features with connections to both the right and left amygdala including the left and right cerebellar tonsils, the left parahippocampal gyrus and the right middle frontal gyrus. Importantly, these features include ROIs spanning the same regions observed in the whole brain voxel‐wise analysis, namely the right parahippocampal gyrus and the right middle frontal gyrus. Thus, taking an ROI‐level approach, the functional connectivity of the left and right amygdala with the same two regions seen in the voxel‐level analyses were found to differentiate individuals with and without a history of I‐ELT. Additionally, the regions showing increased connectivity with the amygdala in the I‐ELT^+^ group span several regions within the frontoparietal network (Yeo et al., 2011).

## 4 Discussion

In the current study, we examined the connectivity of the amygdala during a sustained attention task in groups of Veterans with and without exposure to interpersonal early life trauma, defined as physical/sexual abuse or witnessing family violence before the age of eighteen. Our findings suggest that individuals exposed to I‐ELT show general deficits in sustained attention to stimuli without emotional valence. Specifically, the I‐ELT^+^ group had more lapses and fluctuations of sustained attention, reflected in the lower *d’* scores and increased reaction time variability (CV), respectively. In contrast, factors related to the strategy used by participants (Fortenbaugh et al., 2015), reaction time and criterion, showed no difference between the groups. These observed patterns were significant only when comparing individuals suffering from *interpersonal* types of trauma, as opposed to any traumatic exposure during childhood. This was confirmed by the null finding when comparing the I‐ELT^−^ individuals who reported noninterpersonal trauma before 18 (such as a car accident or natural disaster) with individuals who reported no traumatic exposure before 18 years of age. The observed behavioral deficits in I‐ELT^+^ individuals were accompanied by decreased connectivity between the amygdala and the right parahippocampal gyrus and increased connectivity between the amygdala and the right middle frontal gyrus while participants were engaged in the task. Across all participants, there was a significant association between behavioral performance (d’ and CV) and the strengths of these amygdala connections. Importantly, both the behavioral and amygdala connectivity findings remained significant after controlling for current psychiatric symptoms (e.g., PTSD and depression), age, and number of mTBIs across the lifetime. These results suggest that the present findings were able to isolate unique behavioral and neurobiological deficits in sustained attention associated with a history of I‐ELT. This is notable in that the behavioral and neuroimaging data is collected years and many times decades after the interpersonal traumatic events and can be seen despite potential differences in intervening events that affect cognition and neural functioning (e.g., positive influences such as psychotherapy or education and negative events such as combat exposure or other traumatic events that occur in adulthood).

Collectively, these results extend previous findings of behavioral deficits in sustained attention by highlighting a novel mechanism that may contribute to these deficits, namely, atypical amygdala connectivity patterns with regions normally engaged during sustained attention. While previous studies have suggested that a history of I‐ELT may alter functional connectivity of the amygdala (Philip, Kuras, et al., 2013; Philip, et al., 2013; Philip et al., 2014), this work has been agnostic to the possible cognitive consequences. By demonstrating that altered connectivity patterns can predict behavioral impairments, the present results suggest that how limbic and attention regions communicate can impact fundamental cognitive processes well into adulthood.

Beyond identifying group differences in functional connectivity with the amygdala in circumscribed regions with a mass univariate voxel‐wise approach, we also found, using multivariate modeling, that amygdala connectivity across the entire brain was able to predict individual I‐ELT status with 70% accuracy. While a 70% accuracy rate may not provide a more reliable measure to clinically diagnosing a history of I‐ELT than self‐report, this level of classification accuracy demonstrates that there exists significant brain–behavior relationships wherein a history of I‐ELT is associated with significant alterations in amygdala functional connectivity patterns that are evident years, if not decades, after the traumatic events. Thus, even after accounting for a variety of potential confounding factors such as current PTSD and depression symptoms, there exist significant neurobiological markers of early life trauma that may underlie cognitive deficits in individuals with I‐ELT. Across all participants, the ROIs consistently identified in the classification analysis included the right middle frontal gyrus and parahippocampal gyrus clusters found in the voxel‐level analysis. An additional region was also identified in the left inferior parietal lobe, falling within the frontoparietal control network. This region has been previously found to be engaged during the same sustained attention task (Esterman et al., 2013), suggesting that altered amygdala connectivity in individuals with a history of I‐ELT may broadly impact the network involved in sustained attentional control processes. The lower sensitivity of the multivariate classification analysis compared to the specificity (61% vs. 73%) is consistent with the notion that not every individual exposed to I‐ELT will develop the same atypical amygdala functional connectivity pattern. This may be due to a variety of influences, from genetic factors to age of exposure to the traumatic event. However, the fact that classification reached 70% when we only included 3.5% of the potential brain connections (232/6670 connections for a 116 parcellation) highlights the high probability of amygdala dysfunction in this population, but also leaves open the possibility that functional connectivity patterns between other brain regions could further help to identify individuals with a history of I‐ELT. Indeed, recent analyses of functional connectivity measures during sustained attention tasks have highlighted broad networks of regions outside attention networks that can identify individuals with sustained attention deficits like Attention Deficit Hyperactivity Disorder (Rosenberg et al., 2016). While the present results were able to identify neurobiological markers that are unique to individuals with a history of I‐ELT, future analyses taking into account a broader range of connections across the brain may be able to assess the relationships between a history of I‐ELT, current psychiatric symptoms, and behavioral performance. Additionally, using longitudinal designs it may be possible to use this neurobiological marker of I‐ELT to track behavioral performance trajectories across the lifespan within groups of individuals who all share a history of I‐ELT to determine if this marker is associated with different trajectories. For example, do individuals with a positive history of I‐ELT who also have these atypical amygdala connectivity patterns benefit more or less from cognitive/psychological therapy compared to those without these amygdal connectivity patterns? Do they show accelerated age‐related cognitive declines in sustained attention ability?

With regard to previous studies of I‐ELT and the amygdala, our findings provide a broader framework of the amygdala's role in I‐ELT, particularly highlighting its contribution to poorer nonaffective cognitive performance. Studies of I‐ELT have largely suggested that the impact of stress on development is seen primarily in an affective context (Tottenham et al., 2010). In short, emotional stimuli provoke an increase in the amygdala's reactivity, which overwhelms the inhibitory activity in prefrontal top‐down control regions, resulting in an impaired capacity of individuals to self‐regulate. This mechanism, which echoes the basic neuroanatomy of fear‐conditioning (LeDoux, 2000; Phelps, Delgado, Nearing, & LeDoux, 2004), likely contributes to increased risk for later psychological disorders like PTSD and major depression (Shin & Liberzon, 2010). However, this mechanism of dysregulated amygdala activity has previously been studied without taking into account the often‐reported broader cognitive deficits in executive functions that are seen in many individuals exposed to I‐ELT (van der Kolk, 2003). These impairments in executive function may not be an independent process to the amygdala‐prefrontal emotional loop, but rather be a related process that further increases the risk for adverse outcome after childhood abuse. Because of the nature of our paradigm, this study adds an important dimension to this perspective in showing that the dysregulation of the amygdala is not contingent upon the presence of emotional stimuli, but rather seems independent of the explicit affective nature of the task being performed.

There are at least two potential mechanisms by which the observed amygdala connectivity patterns may impair sustained attention. On the one hand, it may be that the amygdala interferes with normal prefrontal control mechanisms via deregulated salience detection and perceptual filtering (Mitchell et al., 2008). However, we also observed *decreased* connectivity between the amygdala and right parahippocampal gyrus in those with I‐ELT compared to those without. This echoes findings from various studies of abnormal parahippocampal activity associated with PTSD (Francati, Vermetten, & Bremner, 2007). Interestingly, a study by Falconer et al. (2008) investigated inhibitory control in participants with and without PTSD, using a Go/No‐Go task. Their findings showed that healthy participants displayed greater activity of the parahippocampal region compared to individuals with PTSD when contrasting the No‐Go and Go trials. While this suggests that increased activity of the parahippocampal region is beneficial for cognitive performance, other studies have shown that stress may affect the activity of this region and that therapies can help decrease its reactivity (Goldin & Gross, 2010). Thus, the overall activity of this region may fluctuate significantly with the level of stress experienced and the type of task used to probe its behavior.

Finally, it may be that the context of cognitive testing feels more intrinsically stressful, resulting in performance levels beyond the optimal arousal window (Scholz et al., 2009). We note, however, that the tendency to find the present cognitive task more inherently stressful may be due to disruptions in the frontoparietal or limbic systems outlined above. Either way, it appears that, independent of emotional regulation, simple attention skills may already be impaired in individuals with I‐ELT. If this is the case, then it might be beneficial for therapies to include attention training as a foundation for other types of behavior modification (e.g., tonic and phasic attention training; DeGutis & Van Vleet, 2010). Beyond modulating and potentially improving treatment outcomes, sustained attention abilities have been associated with multiple aspects of daily living and functional outcomes including academic achievement (Steinmayr, Ziegler, & Träuble, 2010), driver safety and accidents (Edkins & Pollock, 1997; Yanko & Spalek, 2014), and the ability to develop effective social communication skills (Bennett Murphy, Laurie‐Rose, Brinkman, & McNamara, 2007). Additionally, recent developments have suggested that sustained attention ability may provide a gating mechanism that helps to preserve general cognitive abilities during neurodegeneration associated with aging (Robertson, 2013, 2014; Wilson et al., 2013). Collectively, these findings suggest that above and beyond any emotional disturbances that may be associated with early life trauma, concurrent deficits in sustained attention ability can negatively impact multiple facets of an individual's life throughout the lifespan. Additionally, as psychotherapy is itself a learning‐based process that involves not only cumulative learning but also changing how one interacts with the world, both features that sustained attention is known to be involved with, deficits in sustained attention may prohibit or slow learning and achievement of therapy‐related goals. Thus, addressing sustained attention deficits in addition to emotional and/or psychiatric symptoms may facilitate treatment in these areas.

This study examined performance in a sample of Veterans. While it may be true that Veterans represent a specific subset of the general population, the two samples were well controlled for a variety of factors that may be unique to Veterans, such as combat exposure and number of lifetime mild TBIs. The two samples were additionally matched in terms of current PTSD severity, and we note that I‐ELT has been associated with an increased risk for mental health issues later in life, including PTSD, in a variety of samples that do not share the increased risk for trauma exposure in Veterans. Thus, while the specific type of trauma experienced in adulthood by our Veteran sample (e.g., combat) may be unique, the fact that these participants were exposed to traumatic events as adults is not. Given these factors, the inclusion of Veterans should not limit the generalizability our findings to male non‐Veterans. Furthermore, the TRACTS cohort benefits from an in‐depth psychological assessment that allowed us to include validated clinical measures as covariates in our models.

One limitation of the sample is the low number of females, which is representative of military samples but which prevented us from performing any analyses with sex as a primary factor. Specifically, it must be noted that of the I‐ELT^+^ group, only a single participant was female. While it is possible that the nature of the traumatic experience may have been different depending on the sex of the participant, our analyses do not show significant outliers in PTSD severity or performance that would indicate the presence of two behavioral phenotypes. Most studies of childhood trauma have either examined one specific gender or have reported groups that were uneven, preventing them from conducting gender‐related analyses of the impact of ELT on amygdala structure and function (Tottenham & Sheridan, 2010). However, current research suggests that potential gender differences do exist in sustained attention ability and the strategy used by participants in completing sustained attention tasks. In particular, women have been found to take a more cautious approach to such tasks, with slower overall reaction times (Blatter et al., 2006; Riley et al., 2016). While the cause of these gender differences remains unknown, with some work indicating that cultural factors such a gender inequality may modulate these differences (Riley et al., 2016), it remains an interesting question for future work whether males and females differ in their sustained attention ability independent of early life experiences and whether a history of early life trauma interacts with any potential baseline differences. Additionally, baseline differences in amygdala functional connectivity have been observed across men and women (Kilpatrick, Zald, Pardo, & Cahill, 2006), as well as differences in evoked amygdala activity during an emotional memory task (Cahill et al., 2001). To our knowledge, no work thus far has directly tested for gender differences in amygdala activity on sustained attention or vigilance tasks using nonaffective stimuli. Given the current literature on gender differences in amygdala functioning on affective tasks (Cahill et al., 2001; Killgore, Oki, & Yurgelun‐Todd, 2001; Schneider, Habel, Kessler, Salloum, & Posse, 2000), however, future work including greater numbers of female veterans may help to uncover any potential gender differences in the relationship between amygdala functioning and sustained attention ability.

Additional studies may also help to uncover any potential differences that may exist between the type of I‐ELT experienced, current cognitive functioning, and the gradCPT task. In the present sample, I‐ELT classification was conducted using the TLEQ and included trauma such as physical and sexual abuse. The TLEQ is a validated tool that offers the benefit of covering a wider range of traumatic events that may occur across the lifespan, but it was not developed to solely focus on early life trauma. In its breadth, the TLEQ is limited in the amount of information it provides in terms of abuse‐related traumas. It also does not provide information relative to emotional and physical neglect, which have been shown to impact psychological and biological development (Dannlowski et al., 2012; Hanson et al., 2013; Sheridan, Fox, Zeanah, McLaughlin, & Nelson, 2012). While the TLEQ does not delve into the specifics of types of trauma, it has been shown to be a valid measure for detecting whether or not early life trauma occurred (Clancy et al., 2006; Corbo et al., 2014; Kubany et al., 2000; Van Voorhees et al., 2012). Given this and our sample size, subsamples based on type of I‐ELT were not tested individually. We note, though, that a recent study on the validity of retrospective reports (Hardt & Rutter, 2004) suggests the greatest bias occurs in the details of abuse, not whether abuse occurs, suggesting that the broad level of classification used in the present study (I‐ELT^+^ vs. I‐ELT^−^) is most appropriate given our sample size and population. Considering these factors, we believe that our findings, while limited in terms of the impact of various abuse‐related traumas, do provide consistent and important findings that support greater efforts towards characterizing the cognitive and neural impact of interpersonal trauma in childhood.

In conclusion, this study underscores the lasting negative impact that I‐ELT can have on basic cognitive processes, and demonstrates a relationship between behavioral deficits on a nonemotional sustained attention task and altered amygdala connectivity. The present results further shows the need to explore brain activity with a global perspective that allows detecting relationships between structures that may not have been previously shown using other paradigms. With such an approach, we may be able to develop classification methods that could contribute to the elaboration of new and improved diagnostic tools and interventions.

## Conflict of Interests

The authors declare no competing financial interests.
